# College Museum and Library

**Published:** 1879-10-20

**Authors:** 


### *COLLEGE MUSEUM AND LIBRARY.*

Material for the Southern Medical College Museum and Library is arriving daily. Parties who propose to donate to this good object are informed that beautiful cases are being fitted up for the deposit of their gifts, which will be registered and the name of the donor placed upon every article as a perpetual memento of his generosity.

